# Nutritional Status and Potentially Inappropriate Medications in Elderly

**DOI:** 10.3390/jcm11123465

**Published:** 2022-06-16

**Authors:** Simona Loddo, Francesco Salis, Samuele Rundeddu, Luca Serchisu, Maria Monica Peralta, Antonella Mandas

**Affiliations:** 1 Department of Medical Sciences and Public Health, University of Cagliari, 09124 Cagliari, Italy; simoloddo287@gmail.com (S.L.); samulele83@gmail.com (S.R.); amandas@unica.it (A.M.); 2 University Hospital “Azienda Ospedaliero-Universitaria” of Cagliari, SS554 Bivio Sestu, 09042 Monserrato, Italy; lucaserchisu@gmail.com (L.S.); monicaperalta@virgilio.it (M.M.P.)

**Keywords:** malnutrition, elderly, Mini Nutritional Assessment, inappropriate medications

## Abstract

(1) Background: The association between polypharmacy and malnutrition has been investigated in several studies; however, few of these specifically deepened the relationship between potentially inappropriate medication and malnutrition. With a descriptive approach, the primary aim of our study was to evaluate the impact of the nutritional status, assessed with the Mini Nutritional Assessment (MNA), on potentially inappropriate medications (PIM), estimated 10-year survival, and the risk of adverse drug reactions in elderly patients; the secondary aim was to evaluate how the Screening Tool of Older People’s Prescriptions (STOPP), Screening Tool to Alert to Right Treatment (START), and BEERS 2019 criteria identify PIM compared to nutritional status. (2) Methods: In this study, 3091 subjects were enrolled, of whom 2748 (71.7%) were women; the median age was 80 years, with an interquartile range between 75 and 85 years of age. The subjects were assessed at the outpatient service for frail older people of the University Hospital of Cagliari. The study population was evaluated for their: MNA, Charlson Comorbidity Index, 10-year survival estimation, BEERS 2019, STOPP and START criteria, and ADR Risk scores. (3) Results: We divided the study population into three groups: MNA1 (MNA score ≥ 24), MNA2 (23.5–17), and MNA3 (<17): the severity of comorbidities, STOPP and START alerts, and BEERS 2019 criteria were significantly worse in both MNA2 and MNA3 compared to MNA1—with the exception of BEERS “non-anti-infective medications that should be avoided or have their dosage reduced with varying levels of kidney function in older adults”. Moreover, the estimated 10-year survival was significantly higher in MNA1 than in MNA2 and MNA3, and also in MNA2 compared to MNA3. Finally, the ADR risk scores were significantly lower in MNA1 than in MNA2 and MNA3. (4) Conclusions: Our study demonstrated the association between nutritional status and PIM checked with the BEERS 2019 criteria, and, for the first time, with the STOPP and START criteria.

## 1. Background

Currently, the aging population is a consequence of the increase in life expectancy. In Europe, between 2015 and 2050, the world’s population over 60 years of age will nearly double—from 12% to 22%—with a dissimilar distribution of gender and country [1], and the percentage of people more than 80 years old will quadruple [2]. Until now, living a long life has been associated with a decreased quality of life, mainly due to poor health. Malnutrition is an important problem for the elderly and can cause a deterioration in health. According to the ESPEN (European society for clinical nutrition and metabolism), the Mini Nutritional Assessment (MNA) is the most effective screening tool to evaluate the risk of malnutrition in the elderly [1].

Based on several studies, malnutrition prevalence rates vary from 20% to 30% in clinical settings and from 2% to 8% in community-dwelling older adults, while studies assessing the risk of malnutrition indicate higher rates [2]. Several physiological, socioeconomic, and neuropsychological factors may contribute to insufficient dietary intake and thus lead to malnutrition [2]. In particular, Disease-Related Malnutrition (DRM) is a predominant condition among older people of all health care settings around the world [3]. DRM is associated with chronic morbidity, polypharmacy, higher hospital admission and readmission, and high mortality [3].

Polypharmacy is defined as the use of multiple medications, generally more than five. Older people notoriously have more chronic diseases when compared with younger people, and, consequently, more prescriptions that frequently cause polypharmacy. Clinicians should take care not to prescribe inappropriate medications. Avoidable adverse drug events are serious and important results of potentially inappropriate medication (PIM) use. Polypharmacy and PIM in the elderly are a major public health problem, associated with morbidity and mortality. Therefore, several strategies have been developed to identify PIM: these methods are based on implicit and explicit criteria, themselves based on clinical judgment and consensus for drugs to be avoided, respectively. The implementation of explicit criteria is an important strategy for reducing PIM and adverse drug reactions (ADR): the BEERS criteria, Screening Tool of Older Person’s Prescriptions (STOPP) criteria, Screening Tool to Alert to Right Treatment (START) criteria, and the ADR risk score are the most popular. The BEERS criteria, the most used explicit criteria, were developed by an expert consensus in 1991 and have been revised in 1997, 2003, 2012, 2015, and, most recently, in 2019. The association between polypharmacy and malnutrition has been investigated in numerous studies, and also in various settings [4,5]; however, few of them specifically investigated the relationship between PIM and malnutrition [6]; in particular, loss of appetite, dry mouth, and nausea are common side effects of polypharmacy in the elderly, and they can cause a deterioration in the nutritional status in this population [7].

### Aim of the Study

The primary aim of this descriptive research is to evaluate the impact of nutritional status, assessed with the Mini Nutritional Assessment (MNA), on PIM, estimated 10-year survival, and the risk of adverse drug reactions in elderly patients evaluated in an outpatient setting.

The secondary aim is to evaluate how the STOPP, START, and BEERS 2019 criteria identify PIM compared to nutritional status.

## 2. Materials and Methods

This real-world, cross-sectional study included 3091 first-visit participants aged 65 years or more—of whom 2748 (71.7%) were women—consecutively assessed at the outpatient service for frail older people at the University Hospital of Cagliari from January 2008 to December 2018.

Inclusion criteria: age ≥ 65 years; being subjected to Comprehensive Geriatric Assessment, including MNA.

Exclusion criteria: age < 65 years; not being subjected to MNA.

The enrolled subjects were evaluated, by geriatricians, for:

- MNA: it is a nutritional assessment tool to define the risk of malnutrition; it examines 18 items, divided into four sections (anthropometric—8 points, global—9 points, dietetic—9 points, subjective—4 points). This total score classifies the subject in <17 = malnourished; 17–23.5 = at risk of malnutrition; 24–30 = well-nourished [8].
- Charlson Comorbidity index (CCI): it evaluates the state of health and considers 19 diseases, such as myocardial infarction, angina pectoris or other cardiovascular diseases, dementia, chronic obstructive pulmonary disease (COPD), connective tissue diseases, gastrointestinal diseases, mild or severe liver diseases, diabetes mellitus, stroke, solid and secondary tumours, leukemia, lymphomas, and AIDS. A score between 0 and 6 is assigned to each pathology, and the total score expresses the comorbidity severity index (S). This score is then converted into a 10-year survival estimation by exponential formula: 0.983^A^ (where A = e^S*0.9^) [9,10].
- BEERS 2019 criteria: it is a set of explicit indicators of prescriptive inappropriateness in the elderly. Recorded for each criterion are: the motivation for which the drug is potentially inappropriate, and the recommendation accompanied by the quality of the evidence and the strength of the recommendation. The current criteria include: BEERS for potentially inappropriate medication use in older adults; BEERS for potentially inappropriate medication use in older adults due to drug, disease, or drug–syndrome interaction; BEERS for potentially inappropriate medication to be used with caution in older adults; BEERS for potentially clinically important non-anti-infective drug–drug interactions that should be avoided in older adults; BEERS for non-anti-infective medications that should be avoided or have their dosage reduced with varying levels of kidney function in older adults [11,12].
- STOPP: it consists of a list including 65 indicators of potentially inappropriate drugs divided into 10 clinical–therapeutic areas to facilitate the use of the tool by the prescriber: seven areas belong to different anatomical systems, one area belongs to analgesic drugs, one to drugs that can cause falls, and the last one to duplicate prescriptions [13,14].
- START: it consists of a list of 22 criteria, divided into 6 anatomical systems, which allows identification of underprescription [13,14].
- ADR Risk score: it is a tool that allows the ADR risk to be established. The number of drugs used and the history of previous ADRs are the strongest predictors of ADR, followed by heart failure, the presence of four or more diseases, and renal failure. Each variable corresponds to a score; the sum of all scores, if equal to or greater than 4, defines high risk of adverse reaction [15].

### Statistical Analysis

Quantitative variables were expressed as median and interquartile ranges. Data were analysed using Mann–Whitney test for continuous variables; Kruskal–Wallis test was used to compare the three groups derived from MNA scores; Conover test was performed for post hoc analysis. In order to apply a Logistic Regression, MNA was used as dependent variable, and it was dichotomized so that 0 = MNA < 17, and 1 = MNA ≥ 17; *p*-values > 0.1 were excluded by the model. The results are reported indicating *p*-values in reference to 95% confidence intervals.

Statistical analysis was performed with MedCalc Software Ltd. (version 19.5, Ostend, Belgium).

## 3. Results

The study included 3091 participants, of whom 2215 (71.7%) were women.

The characteristics of the enrolled subjects are shown in Table 1 and Table 2.

A sum of 2544 (82.3%) subjects scored <24 in MNA, of whom 795 scored <17; at least one STOPP and START alert was found in 76.3% and 76.8% of the sample, respectively, and 54.5% and 49% showed at least two STOPP and START alerts, respectively. With regards to the BEERS criteria, 74% of the sample presented at least one alert in “for potentially inappropriate medication use in older adults”, 43.5% in “for potentially inappropriate medication use in older adults due to drug–disease or drug–syndrome interaction”, 62.7% in “for potentially inappropriate medication to be used with caution in older adults”, 10.7% in “for potentially clinically important non-anti-infective drug–drug interactions that should be avoided in older adults”, and 1.2% in “for non-anti-infective medications that should be avoided or have their dosage reduced with varying levels of kidney function in older adults”. Lastly, 1515 subjects (49%) showed an ADR risk score ≥ 4, 51 subjects (1.6%) had 0% estimated 10-years survival, and 701 (22.7%) ≥ 50%.

The analysis showed that the enrolled subjects were taking a median of seven different active principles, and women seemed to take more drugs than men (*p* = 0.002). A sum of 2440 subjects (78.9%) took ≥ 5 medications, and 836 (27%) ≥ 10.

CCI was higher in men than women and, as expected, the estimated 10-year survival was significantly lower in men than in women (*p* < 0.0001) (Table 1).

Furthermore, the number of STOPP alerts was significantly higher in women (median: 2 vs. 1; *p* < 0.0001), while START alerts were higher in men (median: 2 vs. 1; *p* = 0.002) (Table 1).

As regards the BEERS criteria, BEERS “for potentially inappropriate medication use in older adults”, BEERS “for potentially inappropriate medication use in older adults due to drug–disease or drug–syndrome interaction”, and BEERS “for potentially inappropriate medication to be used with caution in older adults” were significantly more frequent in women than in men (Table 1).

We divided the study population based on MNA score into MNA1 (MNA score ≥ 24), MNA2 (23.5–17), and MNA3 (<17): the severity of comorbidities, STOPP and START alerts, and BEERS criteria—with the exception of “non-anti-infective medications that should be avoided or have their dosage reduced with varying levels of kidney function in older adults” (*p* = 0.66)—were significantly worse both in MNA2 and MNA3, compared to MNA1 (*p* < 0.001) (Table 3 and Table 4). In addition, the estimated 10-year survival was significantly higher in MNA1 than in MNA2 and MNA3, and also in MNA2 compared to MNA3 (*p* < 0.0001). Finally, the ADR risk scores were significantly lower in MNA1 than in MNA2 and MNA3 (*p* < 0.0001) (Table 3 and Table 4).

A Stepwise Logistic Regression was performed using the MNA as the dependent variable, and the classes of drugs examined by the BEERS criteria as the independent variables (Table 5). We found that the following classes were significant regressors of MNA: pain medications (*p* = 0.04), benzodiazepines (*p* = 0.0001), proton pump inhibitors (*p* < 0.0001), angiotensin-II-receptor antagonists—sartans (*p* = 0.02), statins (*p* = 0.002), and “other cardiological drugs” (a group containing cardiological drugs except for diuretics, calcium antagonists, beta-blockers, ACE-inhibitors, alpha-blockers, sartans, and antiarrhythmic drugs) (*p* = 0.03). In particular, pain medications, benzodiazepines, proton pump inhibitors, and “other cardiological drugs” showed a coefficient <0, while sartans and statins showed a coefficient >0 (Area Under the Curve: 0.593; 95% confidence interval: 0.575–0.610).

## 4. Discussion

Malnutrition is one of the major problems in the elderly and it can lead to further declines in health and quality of life. It is defined as a state of nutrition that results from the intake or uptake of a lack of nutrients and leads to altered body composition and body cell mass [2].

The most used nutritional state assessing tool is the MNA, presenting high sensitivity and validity for the elderly population [16].

Polypharmacy and use of PIM [6] in older adults are a major public health problem, associated with morbidity and mortality. Moreover, being elderly is associated with metabolic changes and decreased drug clearance, increased drug–drug interactions, prescribing cascades, and greater vulnerability to adverse drug reactions [17].

For these reasons, the aim of this study is the evaluation of the impact of the nutritional status—assessed with MNA—on multimorbidity, estimated 10-year survival, PIM, and the risk of adverse drug reactions in elderly patients evaluated in an outpatient setting.

The noticed prevalence of both malnutrition and the risk of malnutrition is higher than what emerges from the literature [18]: this aspect could be attributed to the fact that the outpatient setting under examination is dedicated to severely impaired elderly. In support of this hypothesis, the median estimated 10-years survival in our sample was 2%.

Considering the two genders, men had more severe comorbidities and a lower estimated 10-year survival, probably due to the prevalence of women in our sample.

The median of the different drugs taken was seven: our study population reflected the most common definition of polypharmacy—“five or more medications daily” [19].

In good agreement with the findings of the international literature [20,21,22,23,24], women showed higher STOPP alerts and lower START alerts.

With regards to the BEERS criteria, they were more frequent in women than in men except for “for non-anti-infective medications that should be avoided or have their dosage reduced with varying levels of kidney function in older adults”, and “for potentially clinically important non-anti-infective drug–drug interactions that should be avoided in older adults”. This may be explained by the fact that women are more likely to search for medical help and express health problems [22]. Moreover, they usually live longer than men and suffer from multiple chronic diseases [21]. The ADR risk score was higher in women as they took a significantly higher number of drugs than men.

Kucukdagli P. et al. [6] showed a correlation between PIM and malnutrition using the BEERS 2012 criteria. In our study this correlation was confirmed using the BEERS 2019 criteria. In particular, by dividing the study population based on MNA score into three groups, well-nourished subjects had a lower frequency of the BEERS criteria compared to subjects at risk and malnourished, except for “for non-anti-infective medications that should be avoided or have their dosage reduced with varying levels of kidney function in older adults”. In this respect, it must be highlighted that only 1.2% of the sample presented this criterion, and this aspect likely justifies the absence of differences in its variance between the groups. Moreover, subjects at risk of malnutrition also had a lower frequency of BEERS criteria compared to the malnourished ones, with the exception of “for potentially clinically important non-anti-infective drug–drug interactions that should be avoided in older adults” and “for potentially inappropriate medication to be used with caution in older adults”. Therefore, the frequency of BEERS criteria—thus of PIM—increased with decreasing MNA scores.

To the best of our knowledge, no previous study has specifically investigated the association between malnutrition and PIM use through STOPP and START alerts. Specifically, in our study, subjects both at risk and malnourished had a greater number of STOPP and START alerts than well-nourished ones; realistically, this is in relation to the greater comorbidity and polypharmacy in subjects with MNA < 24. Consequently, the findings of the BEERS criteria apply equally to STOPP and START: the frequency of these alerts—thus of PIM—likewise increased with the worsening of MNA scores.

This aspect also caused greater ADR risk scores. In addition, well-nourished subjects had lower CCI and better estimated 10-year survival.

The results of the Logistic Regression highlighted the association between malnutrition and PIM, and, in particular, some common classes of drugs. From this multivariate analysis it emerged that pain medications, benzodiazepines, proton pump inhibitors, and “other cardiological drugs” had a negative correlation with malnutrition, that is, their prescription decreases with increasing malnutrition; the opposite happened for sartans and statins.

In conclusion, PIM has to be checked since it is a risk factor for adverse drugs events, and our study confirmed its relationship with nutritional status; this study also showed that the BEERS 2019 criteria are valid tools to show this connection and demonstrated that STOPP and START criteria also are. The limitations of the study are led by its cross-sectional and observational design, which does not allow predictive or causative elements to be drawn; nonetheless, it could be the basis for longitudinal studies describing a causal relationship between nutritional status and polypharmacotherapy, with any intervention strategies.

## Figures and Tables

**Table 1:** Characteristics of the study population.

Patients N.	3091	Gender		Mann–Whitney
		Male N. (%)	Female N. (%)	
		876 (28.3)	2215 (71.7)	
Variables	Median (I.R.)	Median (I.R.)	Median (I.R.)	*p*
Age (years) (Range 65–103)	80 (75–85)	80 (75–85)	80 (76–85)	0.7
**Nutritional Status**				
MNA	20 (17–23)	20.5 (17.5–23.5)	20 (17–22.5)	**0.0001**
**Comorbidities**				
Charlson Comorbidity Index	6 (5–7)	7 (5–8)	6 (4–7)	**<0.0001**
Estimated 10-year survival (%)	2 (0–21)	0 (0–21)	2 (0–53)	**<0.0001**
**Inappropriate Medications Criteria**				
Medications taken (n.)	7 (5–10)	7 (5–9)	7 (5–10)	**0.0019**
STOPP	2 (1–3)	1 (0–3)	2 (1–3)	**<0.0001**
START	1 (1–2)	2 (1–3)	1 (1–2)	**0.0023**
BEERS for potentially inappropriate medication use in older adults	1 (0–2)	1 (0–2)	1 (0–2)	**0.0028**
BEERS for potentially inappropriate medication use in older adults due to drug–disease or drug–syndrome interaction	0 (0–1)	0 (0–1)	0 (0–1)	**<0.0001**
BEERS for potentially inappropriate medication to be used with caution in older adults	1 (0–1)	1 (0–1)	1 (0–1)	**<0.0001**
BEERS for potentially clinically important non-anti-infective drug–drug interactions that should be avoided in older adults	0 (0–0)	0 (0–0)	0 (0–0)	0.38
BEERS for non-anti-infective medications that should be avoided or have their dosage reduced with varying levels of kidney function in older adults	0 (0–0)	0 (0–0)	0 (0–0)	0.54
ADR risk score	3 (2–5)	3 (2–5)	4 (2–5)	0.12

I.R.: interquartile range; MNA: Mini Nutritional Assessment; STOPP: Screening Tool of Older Person’s Prescriptions; START: Screening Tool to Alert to Right Treatment; ADR: adverse drug reaction.

**Table 2:** Distribution of the major comorbidities.

Comorbidities	Percentage
Hypertension	77.2%
Atrial Fibrillation	17.6%
Heart failure	6%
Chronic Cerebrovascular Disease	31.3%
Chronic Obstructive Pulmonary Disease	23.6%
Hepatopathy	16.2%
Chronic Kidney Disease (Cr-Cl <60)	16.4%
Psychiatric Disease (including depression)	35.3%
Diabetes Mellitus	27.9%
Active Neoplasia	10.7%

Cr-Cl: creatinine clearance (CKD-EPI).

**Table 3:** Kruskal–Wallis and MNA: comparison between well-nourished, at risk of malnutrition, and malnourished subjects.

Variables	MNA1	MNA2	MNA3	K-W Test *p*
	MNA ≥ 24	MNA 23.5–17	MNA < 17	
	N. 547 (17.7%)	N. 1873 (60.6%)	N. 671 (21.7%)	
	Median (I.R.)	Median (I.R.)	Median (I.R.)	
Age (years)	79 (74–84)	80 (75–85)	82 (76–86)	**0.0001**
**Comorbidities**				
Charlson Comorbidity Index	6 (4–7)	6 (5–7)	6 (5–8)	**<0.0001**
Estimated 10-year survival (%)	2 (0–53)	2 (0–21)	2 (0–21)	**<0.0001**
**Inappropriate Medications Criteria**				
Medications taken (n.)	6 (4–8)	7 (5–10)	8 (5–10)	**<0.0001**
STOPP	1 (0–2)	2 (1–3)	2 (1–4)	**<0.0001**
START	1 (0–2)	1 (1–3)	2 (1–3)	**<0.0001**
BEERS for potentially inappropriate medication use in older adults	1 (0–1)	1 (0–2)	1 (1–2)	**<0.0001**
BEERS for potentially inappropriate medication use in older adults due to drug– disease or drug–syndrome interaction	0 (0–1)	0 (0–1)	1 (0–1)	**<0.0001**
BEERS for potentially clinically important non-anti-infective drug–drug interactions that should be avoided in older adults	0 (0–0)	0 (0–0)	0 (0–0)	**<0.0001**
BEERS for potentially inappropriate medication to be used with caution in older adults	1 (0–1)	1 (0–1)	1 (0–1)	**<0.0001**
BEERS for non-anti-infective medications that should be avoided or have their dosage reduced with varying levels of kidney function in older adult	0 (0–0)	0 (0–0)	0 (0–0)	0.66
ADR risk score	2 (1–5)	4 (2–5)	5 (2–5)	**<0.0001**

I.R.: interquartile range; MNA: Mini Nutritional Assessment; K-W Test: Kruskal-Wallis Test; STOPP: Screening Tool of Older Person’s Prescriptions; START: Screening Tool to Alert to Right Treatment; ADR: adverse drug reaction.

**Table 4:** Conover Test–MNA: comparison between well-nourished, at risk of malnutrition, and malnourished subjects.

Variables		Average Rank	Different From
Age (years)	MNA1	1395.89	MNA2, MNA3
	MNA2	1539.78	MNA1, MNA3
	MNA3	1685.73	MNA1, MNA2
Charlson Comorbidity Index	MNA1	1368.93	MNA2, MNA3
	MNA2	1533.22	MNA1, MNA3
	MNA3	1679.74	MNA1, MNA2
Estimated 10-year survival	MNA1	1688.5	MNA2, MNA3
	MNA2	1539.48	MNA1, MNA3
	MNA3	1402.17	MNA1, MNA2
Medications taken (n.)	MNA1	1255.71	MNA2, MNA3
	MNA2	1588.49	MNA1
	MNA3	1617.27	MNA1
STOPP	MNA1	1188.27	MNA2, MNA3
	MNA2	1584.24	MNA1, MNA3
	MNA3	1684.06	MNA1, MNA2
START	MNA1	1346.86	MNA2, MNA3
	MNA2	1533.84	MNA1, MNA3
	MNA3	1695.96	MNA1, MNA2
BEERS for potentially inappropriate medication use in older adults	MNA1	1275.24	MNA2, MNA3
	MNA2	1557.47	MNA1, MNA3
	MNA3	1688.15	MNA1, MNA2
BEERS for potentially inappropriate medication use in older adults due to drug–disease or drug–syndrome interaction	MNA1	1287.95	MNA2, MNA3
	MNA2	1562.02	MNA1, MNA3
	MNA3	1665.08	MNA1, MNA2
BEERS for Potentially Clinically Important Non-Anti-infective Drug–Drug Interactions That Should Be Avoided in Older Adults	MNA1	1393.83	MNA2, MNA3
	MNA2	1574.76	MNA1
	MNA3	1543.26	MNA1
BEERS for potentially inappropriate medication to Be Used with Caution in Older Adults	MNA1	1470.63	MNA2, MNA3
	MNA2	1552.75	MNA1
	MNA3	1542.35	MNA1
BEERS for Non-Anti-Infective Medications That Should Be Avoided or Have Their Dosage Reduced with Varying Levels of Kidney Function in Older Adult	MNA1	1533.16	-
	MNA2	1535.74	-
	MNA3	1539.03	-
ADR Risk Score	MNA1	1249.07	MNA2, MNA3
	MNA2	1583.6	MNA1
	MNA3	1636.37	MNA1

MNA: Mini Nutritional Assessment; STOPP: Screening Tool of Older Person’s Prescriptions; START: Screening Tool to Alert to Right Treatment; ADR: adverse drug reaction.

**Table 5:** Logistic Regression MNA vs. classes of drugs.

		MNA	
Variables *	Coefficient	Odds Ratio	*p*
Pain Medications	−0.13	0.81	0.039
Benzodiazepines	−0.29	0.75	0.0001
Proton Pump Inhibitors	−0.37	0.68	<0.0001
Other Cardiological Drugs	−0.21	0.81	0.029
Sartans	0.22	1.25	0.023
Statins	0.31	1.36	0.002

* *p* > 0.01 were excluded by the model.

## Data Availability

The data and materials used and/or analysed during the current study are not publicly available. They are available from the corresponding author on reasonable request.
